# Hemagglutination Assay via Optical Density Characterization in 3D Microtrap Chips

**DOI:** 10.3390/bios13070733

**Published:** 2023-07-14

**Authors:** Sung-Wook Nam, Dong-Gyu Jeon, Young-Ran Yoon, Gang Ho Lee, Yongmin Chang, Dong Il Won

**Affiliations:** 1 Department of Molecular Medicine, School of Medicine, Kyungpook National University, Daegu 41405, Republic of Korea; 2 DanielBio Research Center, Daegu 42694, Republic of Korea; 3 Bio-Medical Research Institute, Kyungpook National University Hospital, Daegu 41940, Republic of Korea; 4 Cell & Matrix Research Institute, Kyungpook National University, Daegu 41944, Republic of Korea; 5 Department of Chemistry, College of Natural Sciences, Kyungpook National University, Daegu 41566, Republic of Korea; 6 Department of Clinical Pathology, School of Medicine, Kyungpook National University, Daegu 41940, Republic of Korea

**Keywords:** hemagglutination, optical density (OD), 3D microtrap chip, 3D printed aperture

## Abstract

Hemagglutination assay has been used for blood typing and detecting viruses, thus applicable for the diagnosis of infectious diseases, including COVID-19. Therefore, the development of microfluidic devices for fast detection of hemagglutination is on-demand for point-of-care diagnosis. Here, we present a way to detect hemagglutination in 3D microfluidic devices via optical absorbance (optical density, OD) characterization. 3D printing is a powerful way to build microfluidic structures for diagnostic devices. However, mixing liquid in microfluidic chips is difficult due to laminar flow, which hampers practical applications such as antigen-antibody mixing. To overcome the issue, we fabricated 3D microfluidic chips with embedded microchannel and microwell structures to induce hemagglutination between red blood cells (RBCs) and antibodies. We named it a 3D microtrap chip. We also established an automated measurement system which is an integral part of diagnostic devices. To do this, we developed a novel way to identify RBC agglutination and non-agglutination via the OD difference. By adapting a 3D-printed aperture to the microtrap chip, we obtained a pure absorbance signal from the microchannels by eliminating the background brightness of the microtrap chip. By investigating the underlying optical physics, we provide a 3D device platform for detecting hemagglutination.

## 1. Introduction

Hemagglutination assay is the agglutination of red blood cells (RBCs) with viruses or antibodies, in which RBCs are used as a source of antigen for the interactions with glycoprotein on the viral surface or the antibodies [1,2,3,4,5,6,7,8]. Recently, researchers developed methods to detect SARS-CoV2 virus by hemagglutination inhibition assay [9,10,11]. Moreover, either hemagglutination or hemagglutination inhibition assays are conventionally used in hospitals for blood typing and for the detection of the influenza virus and bacteria. To extend the assays to general healthcare, various platforms have been developed, including image processing-based detection devices [12,13,14], disposable thermoplastic chips [15,16], and paper-based assay chips [17]. In this regard, portable microfluidic devices for hemagglutination assay are in demand either in hospitals or for point-of-care diagnosis. Despite the broad applications of microfluidics, it is still challenging to connect the newly developed 3D microfluidic chips to the existing benchtop reader machines in order to obtain meaningful information. This report suggests a systematic approach to implement 3D microfluidic chips for detecting hemagglutination via optical density (OD, optical absorbance) characterization by designing a delicate adapting tool such as a 3D printed aperture.

To demonstrate hemagglutination reactions between RBCs and antibodies, we suggest a finely controlled 3D multi-level microfluidic device using a digital light processing (DLP) 3D printer. We call the device a 3D microtrap chip. We note the difficulty of fabricating the multi-level structures using the conventional photolithography-based process due to the costly repeated procedures such as thin-film deposition, pattern generation, and dry or wet etching [18,19,20,21]. Instead, 3D printing efficiently creates multi-level architectures [22,23,24,25,26,27,28,29,30,31,32,33]. One challenge of 3D printing microfluidic devices is the surface roughness caused by nozzle processes or limited by optical resolution, making the fluidic devices leaky [34]. For making the 3D printed surfaces smooth, surface finishing processes such as acetone vapor smoothing have been employed for microfluidic applications [35,36]. Thus far, however, these processes have not been able to prevent the leakage completely. To overcome this issue, we optimized a high-resolution DLP 3D printing method that improves the fabrications of the mold and polydimethylsiloxane (PDMS) chamber, which is an important claim in our fabrication of 3D microfluidic devices.

To implement 3D microfluidic devices as diagnostic tools, mixing reagent solutions with analyte samples, such as whole blood, serum, or plasma, is a prerequisite to induce chemical reactions such as antigen-antibody interaction [37,38,39,40,41,42]. Recent works have demonstrated the possibility of random mixing inside microfluidic chambers, one in which a chaos mixer induces turbulent flow [43,44,45] and another in which a disk-type device generates centrifugal forces to induce mixing between two solutions [46,47,48,49]. Moreover, a variety of microfluidic-based hemagglutination assays with novel detection methods have been developed [50,51,52,53,54,55,56]. In our 3D microtrap chip, the multi-level fluidic structures consist of microwells and microchannels in which two liquid solutions, such as RBCs and antibodies, are uniformly mixed to induce hemagglutination. To validate the 3D microtrap chip as a diagnostic tool, we demonstrated hemagglutination using whole blood samples. Whole blood droplets are trapped in the microwells, which react with the sequentially loaded antibodies to induce hemagglutination.

## 2. Materials and Methods

### 2.1. PDMS Casting for Fabrication of 3D Microtrap Chips

For PDMS casting, the reagents of the Sylgard 184 Silicone elastomer kit (Dow Corning) were mixed with a base-to-curing agent ratio of 10:1. The mixed PDMS reagent solution was poured on the 3D printed ABS mold and degassed in a vacuum desiccator for more than 30 min. The PDMS was cured at 40 °C in an oven overnight. To avoid any thermal deformations of the 3D-printed ABS mold, we performed the PDMS curing process at a low temperature for a long time. After curing, the PDMS upon the 3D printed mold was shaped by a cuter and peeled off from the 3D mold. It was used for the bottom PDMS layer for the microtrap chip. Another PDMS layer was fabricated on flat slide glass for the top layer of the 3D microtrap chip. The inlet and outlet holes were punched using a 1 mm biopsy punch (Kai medical). The surface of both the top and bottom PDMS layers was activated by O_2_ plasma (Femto Science Inc., Plasma processing system CUTE, Hwaseong, Korea) with O_2_ gas 50 sccm with 50 W power. For assembly, the inlet and outlet holes of the top PDMS layer were aligned with the microchannels of the bottom PDMS layer. The assembled PDMS chip was baked at 60 °C in an oven overnight.

### 2.2. Hemagglutination Reactions of Whole Blood Samples in 3D Microtrap Chips

We developed a protocol for whole blood sample loading and antibody mixing. We controlled the flow rates of whole blood and antibody reagents by pipetting. Briefly, 50 μL whole blood was loaded by pushing the pipette plunger “slowly” from the inlet of the 3D microtrap chip for two microchannel lanes. Then, 50 μL whole blood occupied the microchannel. From the outlet, the loaded whole blood was “quickly” pulled by snapping back the pipette plunger to induce a fast flow rate. This process was performed for both lanes. The whole blood droplets were trapped in the microwell region. Antibody reagents were loaded by “slowly” pushing the pipette plunger in order to induce hemagglutination reactions with the trapped whole blood droplets. Then, 50 μL Anti-A was loaded in one lane, while 50 μL anti-B was loaded in the other lane. All procedures were recorded in Supplementary Movie S1.

It is a practical issue to examine the recyclability of the 3D microtrap chips. We carried out the cleaning process by rinsing the microchannels using ethanol 70% solution by manual pipetting. To ensure the removal of residual ethanol, we dried the chip in a convection incubator at 70 °C before reusing the 3D microtrap chip.

## 3. Results

### 3.1. Fabrication of 3D Microtrap Chips

**Figure 1:**
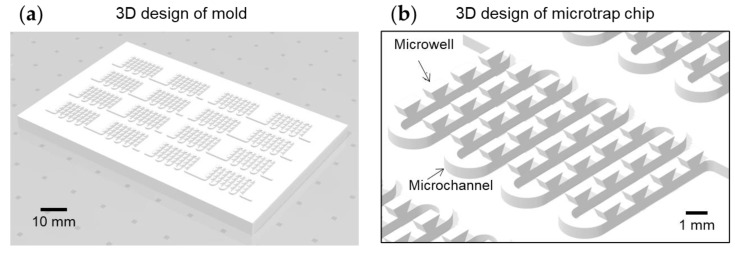
(**a**) 3D design of the mold for microtrap chip. (**b**) The microtrap chip consists of microwell and microchannel geometries.

3D microtrap chips were fabricated using 3D printing-based procedures. We performed 3D structure modeling using computer-aided design (CAD) software (Fusion 360, Autodesk Inc., San Rafael, CA, USA) (Figure 1a). The 3D structure has multi-level geometry. The first level is a meandering microchannel for guiding liquid solutions, and the second level is a microwell for capturing RBC droplets (Figure 1b).

**Figure 2:**
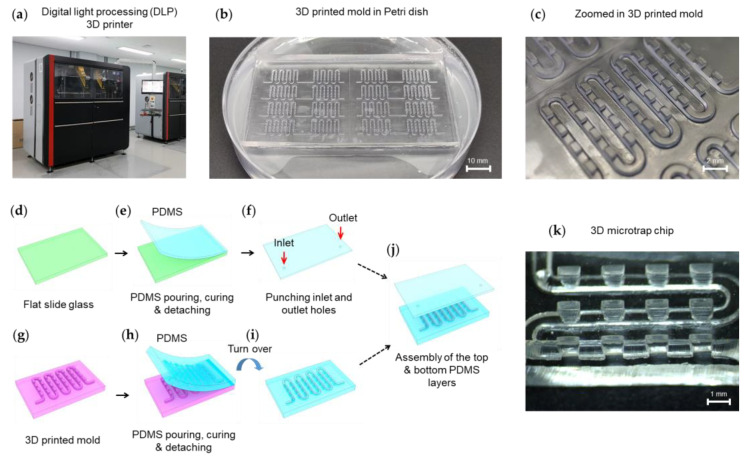
Fabrication of 3D microtrap chips. (**a**) 3D printing of the mold by a digital light processing (DLP) instrument. (**b**) 3D mold structure created by DLP is placed in a 10 cm petri dish for polydimethylsiloxane (PDMS) casting. (**c**) A stereomicroscopic image of the 3D mold with its dimensions of the microchannel and microwell structures. (**d**–**f**) Fabrication procedures of the top layer. On a flat glass slide, a PDMS mixture was poured, cured, and detached. Inlet and outlet holes were punched into the PDMS layer. (**g**–**i**) Fabrication procedures for constructing the bottom layer with multi-level structures consisting of microchannel and microwell. Using the 3D-printed mold, PDMS was cured and detached. (**j**) Two PDMS layers were assembled by plasma treatment. (**k**) Stereomicroscopy image of the tilted cross-section of 3D microtrap chip. The microtrap chip has the specification as follows: microchannel height = 500 µm, microtrap height = 1000 µm, and the ratio of the lower base (500 µm) and upper base = 1:2.

For 3D printing, we employed a DLP instrument (Prodways Tech, ProMaker L5000) in the 3D Convergence Technology Center located in Daegu, Republic of Korea (Figure 2a). The DLP 3D printing machine has 25–150 μm precision. In our design, the minimum feature has 500 μm in the x-y direction and 300 μm in the z-stack. Using the DLP 3D printer, we created a 3D mold of acrylonitrile butadiene styrene (ABS, Prodways Tech, PLASTCure ABS 3650, Montigny-le-Bretonneux, France) (Figure 2b). The ABS resin has a hardness of 84–85 Shore D and a robust tensile modulus of 3.65 GPa. Moreover, it has a high-temperature resistance enough for the PDMS curing process. A stereomicroscope picture shows the multi-level structures of the mold (Figure 2c). The shape of the fabricated 3D-printed mold was transferred to a PDMS layer. 

Two PDMS layers, top and bottom, were assembled (Figure 2d–j). For the top PDMS layer, a PDMS reagent mixture was poured, cured, and peeled off on the flat glass slide (Figure 2d,e). Inlet and outlet holes were generated in the PDMS layer using a biopsy punch (Figure 2f). The 3D printed mold with multi-level structures, including microchannel and microwell, was prepared (Figure 2g). Using the 3D printed mold, the bottom PDMS layer was fabricated (Figure 2h). The PDMS layer has a multi-level structure (Figure 2i). The top and bottom PDMS layers were assembled to construct a 3D microtrap chip (Figure 2j). To investigate the inner structure of the 3D microtrap chip, we cut the PDMS chip along the channel lane, which shows a nice cross-section profile of the microwell (Figure 2k). 

### 3.2. Control of the Reverse Trapezoidal Geometry of the Microwells

We note that the microwell of the microtrap chip has a reverse trapezoidal shape. We note that the RBC emulsion or whole blood has a strong surface tension, thus hindering the separation of the RBC or blood droplet within the microwell region from the flowing stream. To achieve stable droplet formation, we found out that the shape of the microwell plays a crucial role. In particular, the reverse trapezoidal shape has better performance than the vertical sidewall in terms of droplet formation. 

**Figure 3:**
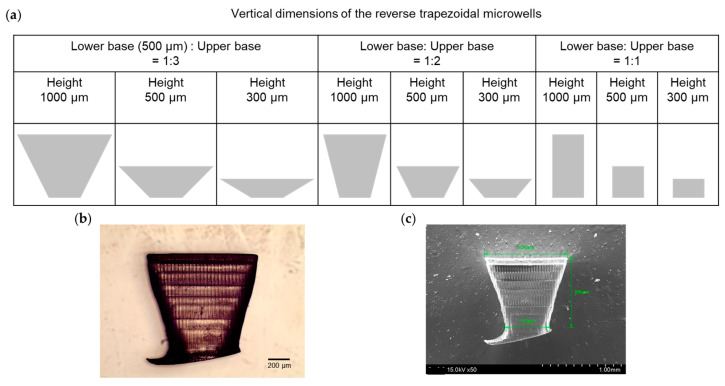
(**a**) Variation of the vertical dimensions of the reverse trapezoidal microwells for nine different 3D printed molds. (**b**) Optical microscope images of the reverse trapezoidal microwell. (**c**) Scanning electron microscopy image of the microwell.

For the fine control over the negative slope of the reverse trapezoidal microwells, we examined a variety of dimensions of the microwell structures for nine 3D-printed molds (Figure 3a). It is a strong benefit of the 3D printing method to control the sidewall of microwells. Briefly, the nine different molds have two variables in the microwell dimensions: One is the ratio of the lower base to the upper base for the reverse trapezoidal shape, such as 1:3, 1:2, and 1:1, and the other is the microwell height, such as 1000 μm, 500 μm, and 300 μm. 

To understand the reverse trapezoidal microwells, we removed a piece of the microwell (lower base (500 µm): upper base = 1:2 with height 1000 µm) from the 3D printed mold. The microwell structure was analyzed using optical microscopy (OM) and scanning electron microscopy (SEM) (Figure 3b,c). The reverse trapezoidal microwells would likely be printed according to the modeling dimension. 

A 3D microtrap chip was produced, which is flexible and optically transparent. Stereomicroscopy images of the cross-section of the 3D microtrap chip, described in Figure 2k, show that the delicate grid structures of the 3D printed ABS mold (Figure 3b,c) for the reverse trapezoidal shapes are well reflected in the PDMS channels.

**Figure 4:**
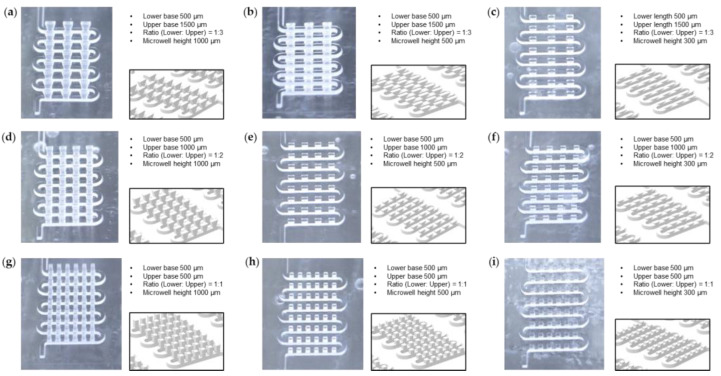
Variation of the dimensions of the microwell structures for 3D microtrap chips. (**a**–**i**) For nine different 3D printed acrylonitrile butadiene styrene (ABS) molds, the details of the dimensions, CAD designs, and stereomicroscopy pictures are summarized. We put two variables as follows: (1) The ratio of the lower base to the upper base of the reverse trapezoidal geometry of the microwell is from 1:3 to 1:1 (row). (2) The microwell height is from 1000 μm to 300 μm (column). Note that all the microwells are designed to have the same lower base, 500 μm, as a constant. Additionally, the microchannels have the same dimensions: The microchannel width is 500 μm, and the microchannel height is 500 μm.

The stereomicroscope pictures of the nine molds with a tilted view clearly show the fine controllability of the 3D printing process for microfluidic structures (Figure 4).

### 3.3. Optical Absorbance Property for the Agglutinated RBCs

**Figure 5:**
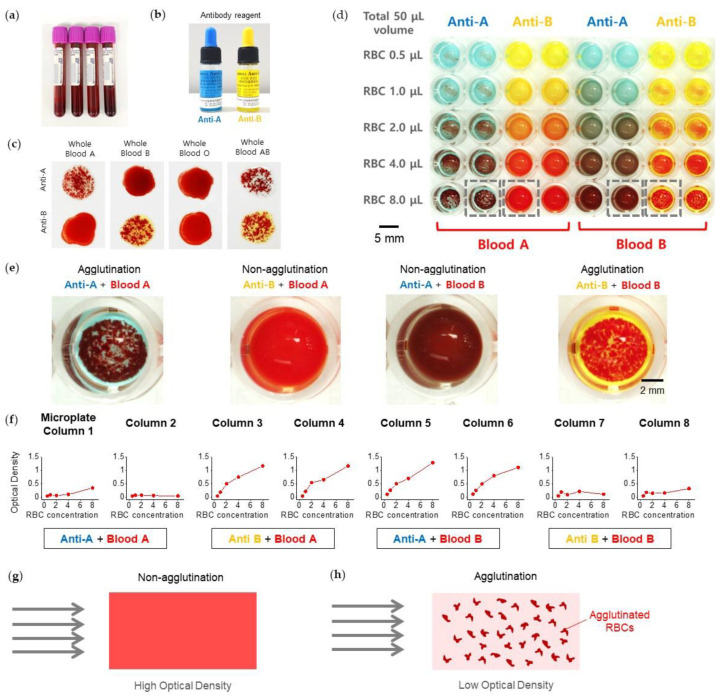
Hemagglutination assay in 3D microtrap chips. (**a**) The whole blood samples are collected in purple top tubes. (**b**) Antibody reagents, anti-A (bluish) and anti-B (yellowish). (**c**) Conventional blood typing test for whole blood A, B, O, and AB. (**d**–**f**) Characterization of optical density (OD, absorbance) for hemagglutination assay in a 96 microwell plate. (**d**) Stereomicroscopy image of a part of a 96-well microplate for hemagglutination assay. (**e**) Magnified images showing the anti-A + blood A and anti-B + blood B for agglutination and the anti-B + blood A and anti-A + blood B for non-agglutination. (**f**) The OD variations as a function of RBC concentration for column 1 to column 8. (**g**,**h**) Diagrams depicting the working principles governing the relationship between agglutination and OD. (**g**) For non-agglutination, high optical density was measured. (**h**) For agglutination, low optical density was measured.

We performed hemagglutination assays using whole blood samples, type A, B, O, and AB. The whole blood samples were collected from healthy donors enrolled in an Institutional Review Board-approved protocol from Kyungpook National University (KNU 2022-0293) (Figure 5a–c). The whole blood samples were treated in purple top blood tubes in which ethylenediaminetetraacetic acid (EDTA) was contained (Figure 5a). Reagents of anti-A and anti-B (Shinyang Chemicals Inc., Busan, Korea) have blue and yellow colors, respectively (Figure 5b). The blood types were checked on a slide glass (Figure 5c).

We characterized the optical absorbance properties for the mixtures of RBCs and antibodies using conventional 96-well plates and a microplate reader (Figure 5d). RBC agglutination was characterized according to the concentration of RBCs in both anti-A (bluish) and anti-B (yellowish). Each combination was tested twice for the four RBC-and-antibody combinations, such as blood A + anti-A, blood A + anti-B, blood B + anti-A, and blood B + anti-B. Each is shown in two columns from column 1 to column 8, as the magnified images of agglutination (anti-A + blood A, anti-B + blood B) and non-agglutination (anti-B + blood A, anti-A + blood B) (Figure 5e). OD variations as a function of RBC concentrations for column 1 to column 8 were plotted (Figure 5f). When RBC agglutination occurs, the OD remains nearly constantly low, as observed for both the anti-A + blood A (columns 1, 2) and anti-B + blood B (columns 7, 8). On the other hand, when RBC agglutination does not occur, the OD significantly increases at the high concentration of RBCs for both the anti-B + blood A (columns 3, 4) and anti-A + blood B (columns 5, 6). Accordingly, we provide a model to describe the dependence of OD variation on the RBC concentration (Figure 5g,h). We assume that RBCs with a 7~8 µm cell size physically block the light path. For non-agglutination, the increase of OD at a high concentration of RBCs is majorly from the uniformly dispersed RBCs (Figure 5g). However, for agglutination, due to the brighter regions besides the agglutinated RBCs, the OD loses its dependence on RBC concentration (Figure 5h). Therefore, the OD remains constantly low.

### 3.4. Hemagglutination in 3D Microtrap Chips

**Figure 6:**
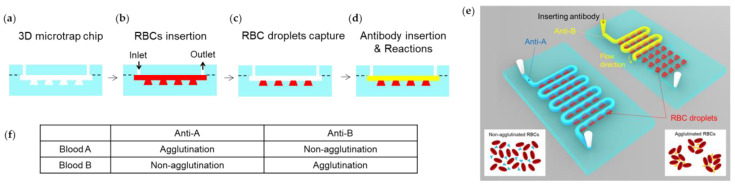
(**a**–**d**) Schematic of red blood cell (RBC) and antibody loading procedures. (**a**) As-fabricated 3D microtrap chip. (**b**) RBC suspension, either blood A or blood B, is inserted into the 3D microtrap chip. (**c**) RBC droplets are captured in the microwells. (**d**) To induce hemagglutination reactions, either anti-A or anti-B are loaded. (**e**) A schematic of hemagglutination assay in 3D microtrap chips. Depending on the surface glycoproteins present on the RBCs, agglutination occurs with corresponding antibodies. Mixing the mismatched RBCs and antibodies does not cause agglutination (lower right). Mixing the matched RBCs and antibodies does cause agglutination (lower left). (**f**) A table summarizing the agglutination and non-agglutination for RBC and antibody mixing.

It is important to mimic the sequence of a diagnostic protocol but consume only a tiny amount of the reagent or sample if small amounts of analyte or reagent are available, such as patient blood, expensive protein, or antibody. In this regard, it is helpful to perform uniform mixing within microfluidic devices instead of vortex mixing, shaking, or pipetting for downstream analysis. We performed a hemagglutination assay using the 3D microtrap chip to examine the solution mixing. We loaded whole blood and antibody reagent to induce hemagglutination inside the 3D microtrap chip (Figure 6). In the microtrap chip, whole blood A or B was loaded (Figure 6a,b). Fast flow rate pulling of whole blood caused droplets to accumulate in the microwells (Figure 6c).

We controlled the flow rate by manual pipetting (Supplementary Movie S1). Then, 50 µL of blood sample was loaded from the inlet by completely filling the channels of the 3D microtrap chip by “slow pushing” the pipette plunger. For trapping the blood droplets, the flow rate should be very high. To induce the high flow rate, a manual “fast pulling” of the pipetting process was performed such that a quick snap back of the pipette plunger at the outlet pulls the loaded blood samples.

Afterward, we added either anti-A or anti-B (Figure 6d). We note that the flow rate for the loading process of antibody reagent should be slow. Normally, it took more than 2–3 s to fill the microchannel with antibody reagent in the 3D microtrap chip by “slow pushing” the pipette plunger. Immediately after loading the antibodies, we monitored the hemagglutination of the mixed whole blood and antibodies in the microchannel and microwell regions.

The surface of the cell membrane of blood cells contains specific glycoproteins that play the role of antigens, classified as type A or type B, thus defining the RBCs as blood A or blood B, respectively. When these antigens bind to specific antibodies, either anti-A or anti-B, RBC agglutinations occur (Figure 6e). However, when the antigens and the antibodies are mismatched, agglutination does not occur. Four different combinations were possible between RBCs and antibodies: Blood A + anti-A, blood A + anti-B, blood B + anti-A, and blood B + anti-B (Figure 6f).

**Figure 7:**
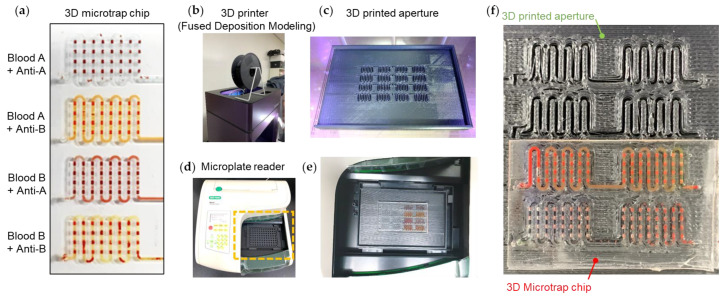
(**a**) Digital camera image of the 3D microtrap chip in which four different mixings occur, corresponding to the blood A + anti-A, blood A + anti-B, blood B + anti-A, and blood B + anti-B. (**b**) A fused deposition modeling (FDM) 3D printer is used for creating a 3D aperture structure. For the FDM process, acrylonitrile butadiene styrene (ABS) filaments are used. (**c**) 3D aperture made by ABS is printed on the bed of the FDM 3D printer. (**d**) A microplate reader machine to measure OD from either a 96-well microplate or a 3D microtrap chip. The orange-dotted region indicates the dock for a 96-well microplate. (**e**) In the orange dotted region of the microplate reader in (**d**), the 3D printed aperture is equipped, upon which the 3D microtrap chip is placed and aligned with the light path. (**f**) The aligned 3D printed aperture and 3D microtrap chip in the microplate reader.

Digital camera image of the 3D microtrap chip in which four combinations of mixing are investigated: Blood A + anti-A, blood A + anti-B, blood B + anti-A, and blood B + anti-B (Figure 7a). RBC agglutinating conditions such as blood A + anti-A and blood B + anti-B make the microchannels bright. In contrast, non-agglutinating conditions such as blood A + anti-B and blood B + anti-A make the microchannels dark. The bright microchannels are caused by the transmitted light beside the agglutinated RBCs. Nevertheless, the dark microchannels are caused by the uniformly dispersed RBCs that block the light path. This working principle inside the 3D microtrap chip agrees with the results from the conventional 96-well microplate, described in Figure 5. 

To develop an automated measurement system for the 3D microtrap chip in conjunction with the benchtop microplate reader, we designed a 3D printed aperture to selectively obtain the signal from microchannels and microwells (Figure 7b–f). It is essential to remove the background brightness outside the microchannel and microwell regions. To eliminate the background brightness, we prepared the aperture using an FDM 3D printer (Rokit, 3dison AEP) (Figure 7b). The FDM process is based on the extrusion of the locally melted ABS filament on the moving bed horizontally and vertically. To efficiently block the background of the 3D microtrap chip, the aperture has an entirely black color with a selectively open microchannel region (Figure 7c). The absorbance characterization (Figure 7d) was performed using a customized iMark microplate reader (Bio-Rad). A broadband white light source, specifically a tungsten halogen lamp, was utilized, with the light passing through the sample. The transmitted light was detected through one of the disk-type filters ranging from 415 nm to 750 nm. Based on the ratio of the intensity of white light and the detected transmitted light, the absorbance values, known as OD, were characterized. The field of view of the detection hole is 18.1 mm^2^ (radius 2.4 mm).

The microplate reader has 96 detector holes (12 × 8) that correspond to the wells of a conventional 96-well microplate. Instead, we substituted the 96-well microplate with the 3D printed aperture and the 3D microtrap chip (Figure 7e). To equip the 3D printed aperture and the 3D microtrap chip together, we first affixed the aperture to the microplate reader. Upon the 3D aperture, we gently placed the 3D microtrap chip by aligning the chip with the detector holes of the microplate reader (Figure 7f).

### 3.5. Hemagglutination Assay via Optical Density Characterization

**Figure 8:**
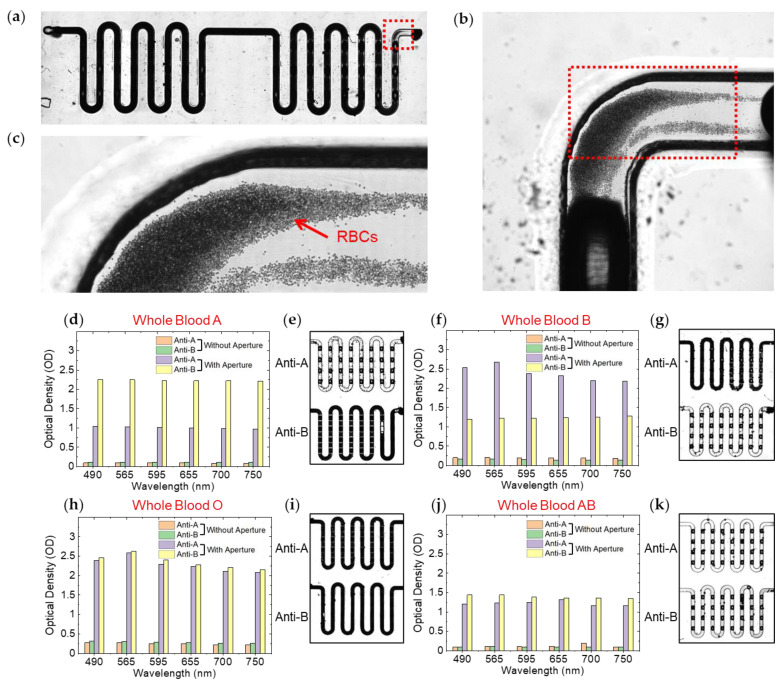
Transmitted light microscopy images of red blood cells (RBCs) (**a**) A meandering microchannel where whole blood is loaded. The microchannel has a dark contrast due to the high concentration of red blood cells (RBCs) in the whole blood. (**b**) A magnified area of the red dotted region in (**a**). (**c**) A magnified area of the red dotted region in (**b**). Individual RBCs are observed. A large number of RBCs contribute a dark contrast (almost black contrast) in the middle of the microchannel. (**d**,**f**,**h**,**j**) Optical density (OD) characterizations for hemagglutination reactions of whole blood A, B, O, and AB with anti-A and anti-B both with and without aperture in a 3D microtrap chip. Low OD values imply agglutinations, while high OD values imply non-agglutinations. (**e**,**g**,**i**,**k**) Transmitted light microscopy images of 3D microtrap chips after hemagglutination reactions were induced by whole blood A, B, O, and AB, with Anti-A and Anti-B. Bright channels imply agglutination, while dark channels imply non-agglutination.

The absorbance originates from the RBCs, which block the light path, making the microchannel dark in the transmitted optic microscopy (Figure 8a–c). We studied the OD variations between RBC agglutination and non-agglutination using the automated OD measurement setup. We measured the OD for different filters ranging in wavelength from 490 nm to 750 nm (Figure 8d,f,h,j). We measured the differences in OD values between agglutination and non-agglutination within the 3D microtrap chip. In particular, before and after equipping the 3D-printed aperture, we identified a pronounced OD difference. Without the aperture, we measured little difference in OD between agglutination and non-agglutination for both whole blood A and B. However, by introducing the 3D-printed aperture, we explicitly identified the difference in OD values.

According to the Lambert–Beer law, OD is expressed as log (I_0_/I), where I_0_ is the intensity of the incident light, and I is the intensity of the transmitted light. For instance, ΔOD = 1 implies ten times in light absorption. Therefore, our measured values of ΔOD, such as 0.8~1.0, are meaningful to differentiate between agglutination and non-agglutination. This finding provides insight into the practical application of the integrated 3D microtrap chip and 3D printed aperture system described here, which becomes a novel platform for the general colorimetric assay.

We correlated the OD values of the 3D microtrap chips (Figure 8d,f,h,j) to the microscopy images (Figure 8e,g,i,k). For transmitted light microscopy observations, we used conventional inverted microscopy (Leica DMi8, Thunder imager, Wetzlar, Germany) for large-area imaging. We obtained 150 to 200 images using a 5× objective lens with a controlled stage by stitching and merging the images to yield a single. 

### 3.6. Optimization of Microwell Specification

By comparing the OD values with the aperture and the large area transmitted light microscopy images, we examined the performance of the 3D microtrap chips in order to obtain the best chip condition (Table 1). To mitigate any experimental bias, we performed repetitions (from 13 to 16 in Table 1) under the same conditions as those in experiments from 1 to 4 in Table 1. Among the conditions of microwell dimensions, we found that the 3D microtrap chip with microwell dimension of height = 300 µm, lower base (500 µm): upper base = 1:1, renders a wrong diagnostic result (see Sample 17 in Table 1). Our opinion is as follows. The 300 µm for microwell depth is the shallowest among our 3D microtrap chip specifications (300, 500, and 1000 µm depth). Moreover, the ratio of lower base and upper base = 1:1 implies the vertical sidewall, which provides more chances to cause errors in performing the assay. On the other hand, when the height is too high, for instance, 1000 µm with a large ratio of lower base and upper base, for instance, 1:3, the structural stability of the reverse trapezoidal microwell geometry of the 3D mold becomes weak, and mechanically fragile, while peeling off the PDMS layer from the 3D printed ABS mold. Therefore, we consider that an intermediate range of microwell height, such as 500 µm with a moderate ratio of lower base and upper base of 1:2, is optimal for the stable operation of a 3D microtrap chip in terms of assay performance and chip fabrication.

### 3.7. Statistical Analysis of the Hemagglutination Assay in 3D Microtrap Chips 

**Figure 9:**
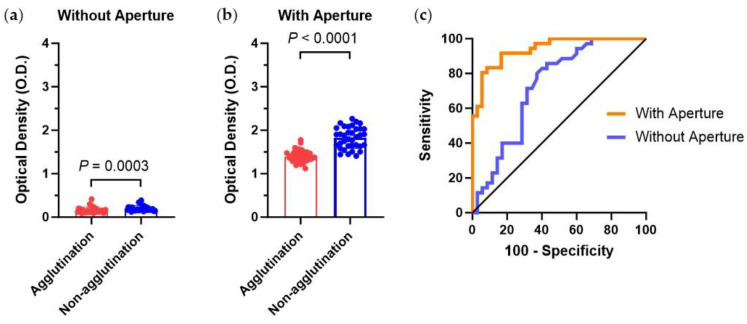
(**a**,**b**) For the hemagglutination assay (n = 140) of agglutination and non-agglutination from whole blood A, B, O, and AB, with Anti-A and Anti-B in the best conditioned 3D microtrap chips such as a microwell height of 500 µm with a ratio of lower base and upper base of 1:2. OD values were measured both without (**a**) and with (**b**) aperture. (**c**) Receiver operating characteristic curves are analyzed for OD values both with and without aperture.

Based on the optimization of the chip conditions, we obtained the best chip conditions, such as the ratio of lower base to upper base as 1:2 with a 500 µm height. Using this condition of the 3D microtrap chip, we statistically analyzed the hemagglutination reactions of whole blood A, B, O, and AB with Anti-A and Anti-B (Figure 9). 

For the detection of hemagglutination assay in the 3D microtrap chips, we statistically analyzed the assay (n = 140) in the best optimized 3D microtrap chip without and with aperture (Figure 9a,b, and Supplementary Table S1). Based on the results of the OD values, we obtained the receiver operating characteristic (ROC) curve to analyze the area under the curve (AUC) using Prism software (GraphPad) (Figure 9c). Without the aperture, AUC, sensitivity, and specificity are 0.726, 71.43%, and 68.57%, respectively. By introducing the 3D-printed aperture, the diagnostic accuracy was significantly improved with AUC, sensitivity, and specificity of 0.939, 83.33%, and 83.33%, respectively.

## 4. Discussion

The 3D printing process has become widely adopted for both educational and advanced research purposes. In the field of microfluidic applications, traditional methods such as semiconductor-based processes were utilized to fabricate microfluidic devices for biomedical applications, including diagnostics and therapeutics [57,58]. For instance, SU8 molding is one of the popular methods for PDMS chamber fabrication [18]. Moreover, various micro and nano-size patterns on silicon wafers have served as molds [19]. Additionally, microfluidic structures have been achieved using materials such as silicon, metal, and oxide [20]. However, these technologies are limited to the researcher, who can access contact aligners or precision machining. Recent advancements in 3D printing have revolutionized the fabrication of microfluidic devices, providing opportunities for a wider range of researchers who are interested in biomedical devices. The simplicity and convenience of 3D printing offer a significant advantage in creating microfluidic structures. Therefore, a simple and convenient method to create microfluidic structures via 3D printing is essential. Moreover, connecting the 3D printing-based fluidic device to practical biomedical applications is another challenge.

The resolution or precision of the 3D printing process is a critical factor to consider. Fused deposition modeling (FDM) printers such as the Prusa 3D printer (Czech Republic), Ateam Ventures 3D printer (Ateam company, Korea), and Edison AEP printer (Rokit company, Korea), are more affordable, ranging from $100 to 1000 price, and are suitable for achieving resolutions of 100 μm or larger [33,59,60]. However, based on our experience, FDM-printed molds often result in severe leakage issues due to the surface roughness of the printed mold [34,35,36]. To address this challenge, we employed stereolithography (SLA) or digital light processing (DLP) printers such as the Form 3L printer (Formlabs, Somerville, Massachusetts, USA) and ProMaker L5000 (Prodways Tech, France) to fabricate molds for microfluidic applications, allowing us to achieve resolutions of 10–100 μm [30]. The equipment cost for these SLA/DLP printers is around $10,000 for the commercialized Formlabs, which may be considered relatively expensive but provides high efficiency and performance. In our mold fabrication experiments for microfluidics, we tested various equipment, including FDM, DLP, and SLA. DLP and SLA printers showed more promising results for 3D microfluidic molds, ensuring stable fluidic connections (Figure 2 and Figure 7) and exhibiting large-area printing capabilities with reasonable printing yield [30].

Furthermore, recent advancements in SLA printers, such as micro stereolithography printers, e.g., the Boston Micro Fabrication printer (BMF, Maynard, Massachusetts, USA), have introduced fine lens modules, enabling sub-10 μm resolution with improved accuracy and precision compared to conventional SLA printers [61,62]. However, the high cost of BMF equipment, exceeding $100,000, may pose a significant barrier. Alternatively, in our case (not described in this report), we opted to utilize mold printing services, which cost only a few hundred dollars, making it more feasible for conventional 3D printing researchers and users. However, it should be noted that the micro stereolithography method is not practical for large-scale fabrication of microfluidic molds due to limited printing yield. Instead, the BMF-printed molds can be employed to mimic the extremely fine surface of organs, skin, and tissue, in a small area [61]. 

To meet the condition of simple 3D printing technology in conjunction with biomedical diagnosis devices, our report suggests the implementation of hemagglutination assay in 3D printing-based microfluidics as one approach to bridge the gap between 3D printing technology and medical diagnosis. Both hemagglutination and hemagglutination inhibition assay are well-established diagnostic methods used for the detection of viruses, pathogens, and blood typing [1,2,3,4,5,6,7,8,9,10,11]. The demonstration of the hemagglutination assay in our study represents an interesting approach. Notably, we introduce the adoption of an optical method for detecting hemagglutination, which is a novel aspect of our manuscript.

Recently, due to advanced imaging techniques, the applications of biomedical devices become versatile. The detection of hemagglutination in the 3D microtrap chip was based on light absorbance detection. However, the novel detecting method can be extensively used in more sensitive and accurate ways, such as interferometric scattering microscopy, SPR, or LSPR-based imaging [12]. In addition, a number of sensing methods have been developed to monitor the formation of agglutinated RBCs [50,51,52,53,54,55,56]. Ferraz et al. suggested an image processing technique using a CCD camera to distinguish between agglutination and non-agglutination [13]. Moreover, Hueta et al. suggested a passive microfluidic biochip for automated measurement of RBCs agglutination by image process [14]. 

Integrating the 3D microtrap chip with advanced imaging processes can be a plausible way, which can potentially extend its applications for point-of-care diagnostics. To connect them, it is essential to develop adapters to compensate for the gap between the chip and the imaging system. 3D-printed adapters were employed for optical connection and fluorescence detection [63,64,65]. In our method, we suggest a 3D-printed aperture as one of the adapters to link the 3D-based microfluidic device with a conventional absorbance-based microplate reader machine. The 3D-printed aperture described here can be diversely utilized for a wide range of optics, such as fluorescence and luminescence-based detection as well as absorbance. Most of all, the adapter technology, such as the aperture, enables smartphone-based detection of various biomedical events, such as antibody-antigen interaction and biotin-streptavidin, which are widely used in molecular biology assays [63,64]. We believe that our 3D printing-based adapter approach will inspire biomedical researchers to explore novel platforms for applying their biomedical assays.

Our approach, combining simple 3D printing, hemagglutination assay, and optical detection, is a versatile platform. We discovered that the absorbance originates from the RBCs blocking the light path, resulting in a dark microchannel in transmitted light microscopy. To ensure the reliability and significance of our method, statistical treatments of multiple cases are crucial for clinical applicability. By analyzing ROC curves, we estimate the performance of our 3D microtrap chip in which the AUC is higher than 0.9 with sensitivity and specificity both higher than 80%. This analysis highlights the potential of our 3D printing-based prototype medical device to provide valuable information about diagnostic accuracy.

## 5. Conclusions

In conclusion, our study presents a comprehensive and systematic approach to developing a 3D printing-based diagnostic device. We have addressed various aspects, including fabrication techniques, recycling methods, antigen-antibody-based hemagglutination assay, optical detection, and adapter technology, along with statistical validation. We began our research by carefully selecting 3D printers suitable for microfluidic applications, in which SLA and DLP printing methods are practically suitable for mold fabrication with high yields. Notably, our 3D-printed molds feature unique reverse trapezoidal shapes, which play a vital role in effectively trapping blood droplets. To show the feasibility of the 3D printing-based microfluidic device, we conducted a hemagglutination assay for blood typing. It is important to highlight that the detection of hemagglutination in the 3D microtrap chip relies on the fundamental optical absorbance resulting from the light-blocking behavior of red blood cells (RBCs). Additionally, we address the significance of adapter technology in bridging the gap between biomedical devices and optical equipment. We present a 3D printed aperture to obtain a pure absorbance signal from hemagglutination in the 3D microtrap chip, enabling the usage of the conventional microplate reader in conjunction with the 3D biomedical device. Statistical analyses, such as ROC curve for evaluating the AUC, sensitivity, and specificity, are a meaningful approach to validating the biomedical device. 

The implications of our research extend to the realm of point-of-care testing, where our technology holds the potential for the rapid and accurate detection of viruses, bacteria, and pathogens. Furthermore, our approach holds educational value, serving as a valuable tool for teaching and learning. We firmly believe that the fusion of 3D printing and biomedical assay technology is highly attractive to biomedical researchers seeking to develop innovative platforms for biochemical assays.

## Figures and Tables

**Table 1:** Optimization of the conditions for the 3D microtrap chips.

Sample#	Microwell Dimension		Whole Blood Type	Optical Density (OD)						Diagnostic Decision	⭘/🞩
				Without Aperture		With Aperture					
	Height (μm)	Lower Base (500 μm): Upper Base		Anti-A	Anti-B	Anti-A	<1.7 (Agglutination) >1.7 (Non-agglutinaion)	Anti-B	<1.7 (Agglutination) >1.7 (Non-agglutinaion)		
1	500	1:2	A	0.148	0.194	1.256	Agglutination	2.146	Non-agglutination	A	⭘
2	500	1:2	B	0.348	0.228	1.875	Non-agglutination	1.233	Agglutination	B	⭘
3	500	1:2	O	0.210	0.286	2.555	Non-agglutination	2.638	Non-agglutination	O	⭘
4	500	1:2	AB	0.164	0.120	1.258	Agglutination	1.338	Agglutination	AB	⭘
5	500	1:3	A	0.165	0.214	1.504	Agglutination	1.881	Non-agglutination	A	⭘
6	500	1:3	B	0.150	0.136	2.134	Non-agglutination	1.349	Agglutination	B	⭘
7	500	1:3	O	0.337	0.382	2.313	Non-agglutination	2.486	Non-agglutination	O	⭘
8	500	1:3	AB	0.240	0.223	1.119	Agglutination	1.529	Agglutination	AB	⭘
9	300	1:2	A	0.139	0.183	1.341	Agglutination	1.928	Non-agglutination	A	⭘
10	300	1:2	B	0.186	0.115	1.959	Non-agglutination	1.288	Agglutination	B	⭘
11	300	1:2	O	0.198	0.232	3.002	Non-agglutination	3.500	Non-agglutination	O	⭘
12	300	1:2	AB	0.254	0.153	0.936	Agglutination	1.169	Agglutination	AB	⭘
13	500	1:2	A	0.124	0.160	1.211	Agglutination	2.008	Non-agglutination	A	⭘
14	500	1:2	B	0.292	0.148	1.837	Non-agglutination	1.230	Agglutination	B	⭘
15	500	1:2	O	0.247	0.323	2.058	Non-agglutination	1.810	Non-agglutination	O	⭘
16	500	1:2	AB	0.159	0.204	1.214	Agglutination	0.979	Agglutination	AB	⭘
17	300	1:1	A	0.126	0.127	1.004	Agglutination	1.170	Agglutination	O	🞩
18	300	1:1	B	0.244	0.195	2.421	Non-agglutination	1.113	Agglutination	B	⭘
19	300	1:1	O	0.197	0.190	2.184	Non-agglutination	2.164	Non-agglutination	O	⭘
20	300	1:1	AB	0.144	0.176	1.289	Agglutination	1.381	Agglutination	AB	⭘
21	1000	1:2	A	0.096	0.107	1.010	Agglutination	2.232	Non-agglutination	A	⭘
22	1000	1:2	B	0.198	0.151	2.383	Agglutination	1.238	Agglutination	B	⭘
23	1000	1:2	O	0.247	0.284	2.284	Non-agglutination	2.355	Non-agglutination	O	⭘
24	1000	1:2	AB	0.120	0.098	1.220	Agglutination	1.390	Agglutination	AB	⭘

## Data Availability

The data presented in this study are available on request from the corresponding author. The data are not publicly available due to agreement of confidentiality.

## Supplementary Materials

The following supporting information can be downloaded at: https://www.mdpi.com/article/10.3390/bios13070733/s1, Table S1: Statistical analysis of hemagglutination assay in 3D microtrap chips; Movie S1: Hemagglutination assay in 3D microtrap chip.
