# Supplementary material for: Hemagglutination Assay via Optical Density Characterization in 3D Microtrap Chips
Source: Biosensors (Basel). 2023 Jul 14;13(7):733. doi: 10.3390/bios13070733 (PMC10377501; doi:10.3390/bios13070733)
Supplement: Supplementary file 1 [file biosensors-13-00733-s001.zip › supplementary S2.pdf]

Supplementary Materials

Hemagglutination Assay via Optical Density Characterization in 3D Microtrap Chips

Sung-Wook Nam[1,2,3,*], Dong-Gyu Jeon[1,4], Young-Ran Yoon[1], Gang Ho Lee [5], Yongmin Chang[1] and

Table S1. Statistical Analysis of Hemagglutination Assay in 3D Microtrap Chips

With Aperture						Without Aperture					
Agglutination			Non-agglutination			Agglutination			Non-agglutination		
A	Anti A	1.287	A	Anti B	2.168	B	Anti B	0.112	B	Anti A	0.164
B	Anti B	1.375	B	Anti A	1.504	AB	Anti A	0.135	O	Anti A	0.232
AB	Anti A	1.319	O	Anti A	1.509	AB	Anti B	0.19	O	Anti B	0.239
AB	Anti B	1.416	O	Anti B	1.633	B	Anti B	0.236	B	Anti A	0.341
B	Anti B	1.368	B	Anti A	1.903	B	Anti B	0.167	B	Anti A	0.184
B	Anti B	1.295	B	Anti A	1.701	A	Anti A	0.271	A	Anti B	0.245
A	Anti A	1.24	A	Anti B	1.541	A	Anti A	0.115	A	Anti B	0.228
A	Anti A	1.123	A	Anti B	1.448	AB	Anti B	0.131	O	Anti B	0.158
AB	Anti B	1.301	O	Anti B	1.771	AB	Anti B	0.102	O	Anti B	0.14
AB	Anti B	1.234	O	Anti B	1.887	AB	Anti A	0.114	O	Anti A	0.265
AB	Anti A	1.328	O	Anti A	1.637	AB	Anti A	0.104	O	Anti A	0.218
AB	Anti A	1.195	O	Anti A	2.051	A	Anti A	0.14	A	Anti B	0.161
A	Anti A	1.23	A	Anti B	1.407	A	Anti A	0.091	A	Anti B	0.131
A	Anti A	1.217	A	Anti B	1.441	B	Anti B	0.26	B	Anti A	0.172
B	Anti B	1.595	B	Anti A	2.198	B	Anti B	0.111	B	Anti A	0.123
B	Anti B	1.339	B	Anti A	1.654	AB	Anti B	0.151	O	Anti B	0.161
AB	Anti B	1.476	O	Anti B	1.749	AB	Anti B	0.09	O	Anti B	0.164
AB	Anti B	1.474	O	Anti B	1.878	AB	Anti A	0.155	O	Anti A	0.153
AB	Anti A	1.57	O	Anti A	1.58	AB	Anti A	0.15	O	Anti A	0.182
AB	Anti A	1.492	O	Anti A	1.796	A	Anti A	0.201	A	Anti B	0.215
A	Anti A	1.468	A	Anti B	2.084	A	Anti A	0.189	A	Anti B	0.184
A	Anti A	1.343	A	Anti B	2.034	B	Anti B	0.126	B	Anti A	0.168
B	Anti B	1.5	B	Anti A	2.028	B	Anti B	0.089	B	Anti A	0.138
B	Anti B	1.383	B	Anti A	1.949	AB	Anti B	0.161	O	Anti B	0.232
AB	Anti B	1.559	O	Anti B	1.908	AB	Anti B	0.118	O	Anti B	0.225
AB	Anti B	1.447	O	Anti B	1.91	AB	Anti A	0.117	O	Anti A	0.168
AB	Anti A	1.479	O	Anti A	1.937	AB	Anti A	0.141	O	Anti A	0.157
AB	Anti A	1.357	O	Anti A	2.027	A	Anti A	0.149	A	Anti B	0.18
A	Anti A	1.408	A	Anti B	1.662	A	Anti A	0.143	A	Anti B	0.164
A	Anti A	1.329	A	Anti B	1.662	B	Anti B	0.414	B	Anti A	0.328
B	Anti B	1.711	B	Anti A	2.116	B	Anti B	0.226	B	Anti A	0.286
B	Anti B	1.369	B	Anti A	2.16	AB	Anti B	0.302	O	Anti B	0.171
AB	Anti B	1.544	O	Anti B	2.091	AB	Anti B	0.157	O	Anti B	0.388
AB	Anti B	1.413	O	Anti B	2.264	AB	Anti A	0.204	O	Anti A	0.149
AB	Anti A	1.774	O	Anti A	1.648	AB	Anti A	0.162	O	Anti A	0.331
AB	Anti A	1.379	O	Anti A	1.844						
